# Supplementary material for: Seed tuber imprinting shapes the next-generation potato microbiome
Source: Environ Microbiome. 2024 Feb 21;19:12. doi: 10.1186/s40793-024-00553-w (PMC10882817; doi:10.1186/s40793-024-00553-w)
Supplement: Supplementary file 2 — Additional file 2. Supplementary Tables 1–7. [file 40793_2024_553_MOESM2_ESM.pdf]

Table S1 Results of Permutational multivariate analysis (PERMANOVA) for variance of the bacterial composition for seed tuber, post-storage seed tuber, daughter tuber and root samples at ASV level. Tests are based on Bray-Curtis dissimilarity distances and 999 permutations.

Sample Type	Factors	Degrees of Freedom	Sums of Squares	Mean Squares	F.Model	R^2	*P*
All Samples	Generation	1	17.33	17.33	99.47	0.25	0.001
	Sample Type	2	10.65	5.32	30.55	0.15	0.001
	Residuals	239	41.63	0.17	NA	0.60	NA
	Total	242	69.60	NA	NA	1.00	NA
Seed Tuber	Variety	1	0.77	0.77	14.09	0.18	0.001
	Production Field	4	2.79	0.70	12.78	0.64	0.001
	Residuals	15	0.82	0.05	NA	0.19	NA
	Total	20	4.38	NA	NA	1.00	NA
Post-storage Seed Tuber	Variety	1	1.40	1.40	19.93	0.17	0.001
	Production Field	4	4.65	1.16	16.55	0.57	0.001
	Residuals	29	2.04	0.07	NA	0.25	NA
	Total	34	8.08	NA	NA	1.00	NA
Daughter Tuber	Variety	1	1.54	1.54	8.67	0.08	0.001
	Production Field	4	1.32	0.33	1.86	0.07	0.001
	Residuals	89	15.85	0.18	NA	0.85	NA
	Total	94	18.72	NA	NA	1.00	NA
Root	Variety	1	0.36	0.36	3.36	0.03	0.001
	Production Field	4	0.82	0.20	1.88	0.08	0.001
	Residuals	86	9.34	0.11	NA	0.89	NA
	Total	91	10.52	NA	NA	1.00	NA

Table S2 Results of PERMANOVA for variance of the fungal composition for seed tuber, post-storage seed tuber, daughter tuber and root samples at ASV level. Tests are based on Bray-Curtis dissimilarity distances and 999 permutations.

Sample Type	Factors	Degrees of Freedom	Sums of Squares	Mean Squares	F.Model	R^2	*P*
All Samples	Generation	1	14.58	14.58	72.43	0.23	0.001
	Sample Type	2	5.25	2.63	13.04	0.08	0.001
	Residuals	216	43.48	0.20	NA	0.69	NA
	Total	219	63.32	NA	NA	1.00	NA
Seed Tuber	Variety	1	0.71	0.71	8.28	0.17	0.001
	Production Field	4	2.33	0.58	6.85	0.55	0.001
	Residuals	14	1.19	0.09	NA	0.28	NA
	Total	19	4.23	NA	NA	1.00	NA
Post-storage Seed Tuber	Variety	1	0.77	0.77	5.33	0.10	0.001
	Production Field	4	3.67	0.92	6.34	0.46	0.001
	Residuals	25	3.61	0.14	NA	0.45	NA
	Total	30	8.05	NA	NA	1.00	NA
Daughter Tuber	Variety	1	0.60	0.60	2.85	0.03	0.001
	Production Field	4	1.66	0.42	1.97	0.10	0.001
	Residuals	71	15.01	0.21	NA	0.87	NA
	Total	76	17.28	NA	NA	1.00	NA
Root	Variety	1	0.72	0.72	5.01	0.05	0.001
	Production Field	4	0.97	0.24	1.69	0.07	0.001
	Residuals	86	12.35	0.14	NA	0.88	NA
	Total	91	14.04	NA	NA	1.00	NA

Table S3 Results of PERMANOVA test of bacterial and fungal composition of seed tuber and post-storage seed tuber samples at ASV level. Tests are based on Bray-Curtis dissimilarity distances and 999 permutations with FDR correction for multiple comparisons.

	Sample Type 1	Sample Type 2	Sample Size	Permuations	pseudo-F	p-value	q-value
Bacteria	Seed Tuber	Post-storage Seed Tuber	56	999	4.223	0.001	0.001
Fungi	Seed Tuber	Post-storage Seed Tuber	51	999	2.345	0.019	0.019

Table S4 Results of PERMANOVA test of bacterial and fungal composition of different sample types at ASV level. Tests are based on Bray-Curtis dissimilarity distances and 999 permutations with FDR correction for multiple comparisons.

	Sample Type 1	Sample Type 2	Sample Size	Permuations	pseudo-F	p-value	q-value
Bacteria	Seed Tuber	Daughter Tuber	116	999	38.908	0.001	0.001
	Seed Tuber	Root	113	999	59.571	0.001	0.001
	Daughter Tuber	Root	187	999	61.369	0.001	0.001
Fungi	Seed Tuber	Daughter Tuber	97	999	24.680	0.001	0.001
	Seed Tuber	Root	112	999	44.832	0.001	0.001
	Daughter Tuber	Root	169	999	24.923	0.001	0.001

Table S5 Results of pairwise Adonis test of bacterial composition of 5 tuber compartments at ASV level. Tests are based on Bray-Curtis dissimilarity distances and 999 permutations with FDR correction for multiple comparisons.

Group 1	Group 2	Sample size	Permutations	pseudo-F	p-value	q-value
Adhering soil	Heel end	45	999	7.926	0.001	**0.002**
Adhering soil	Eye	36	999	4.250	0.001	**0.002**
Adhering soil	Peel	47	999	4.183	0.001	**0.002**
Adhering soil	Flesh	34	999	2.210	0.002	**0.003**
Heel end	Eye	31	999	1.322	0.055	0.061
Heel end	Peel	42	999	2.479	0.001	**0.002**
Heel end	Flesh	29	999	3.091	0.001	**0.002**
Eye	Peel	33	999	1.243	0.143	0.143
Eye	Flesh	20	999	1.592	0.012	**0.015**
Peel	Flesh	31	999	1.546	0.009	**0.013**

Table S6 Results of pairwise Adonis test of fungal composition of 5 tuber compartments at ASV level. Tests are based on Bray-Curtis dissimilarity distances and 999 permutations with FDR correction for multiple comparisons.

Group 1	Group 2	Sample size	Permutations	pseudo-F	p-value	q-value
Adhering soil	Eye	44	999	3.409	0.001	**0.002**
Adhering soil	Flesh	35	999	2.849	0.003	**0.004**
Adhering soil	Heel end	49	999	10.021	0.001	**0.002**
Adhering soil	Peel	47	999	4.386	0.001	**0.002**
Eye	Flesh	31	999	1.929	0.030	**0.033**
Eye	Heel end	45	999	2.976	0.002	**0.003**
Eye	Peel	43	999	0.614	0.830	0.830
Flesh	Heel end	36	999	5.041	0.001	**0.002**
Flesh	Peel	34	999	2.584	0.011	**0.014**
Heel end	Peel	48	999	4.351	0.001	**0.002**

Table S7 Results of pairwise Adonis test of bacterial and fungal composition of sprout and 5 tuber compartments at ASV level. Tests are based on Bray-Curtis dissimilarity distances and 999 permutations with FDR correction for multiple comparisons.

	Sample Type 1	Sample Type 2	Sample Size	Permuations	pseudo-F	p-value	q-value
Bacteria	Adhering soil	Sprout	65	999	23.955	0.001	0.001
	Eye	Sprout	60	999	19.134	0.001	0.001
	Flesh	Sprout	56	999	13.605	0.001	0.001
	Heel end	Sprout	64	999	23.609	0.001	0.001
	Peel	Sprout	64	999	21.230	0.001	0.001
Fungi	Adhering soil	sprout	75	999	19.990	0.001	0.002
	Eye	sprout	70	999	14.987	0.001	0.002
	Flesh	sprout	62	999	13.753	0.001	0.002
	Heel end	sprout	76	999	25.635	0.001	0.002
	Peel	sprout	74	999	17.470	0.001	0.002
